# The pattern and spread of invasion can predict late cervical lymph node metastasis in early tongue squamous cell carcinoma

**DOI:** 10.1186/s13000-023-01371-3

**Published:** 2023-08-03

**Authors:** Koroku Kato, Hiroki Miyazawa, Hisano Kobayashi, Yoshiaki Kishikawa, Hayato Funaki, Natsuyo Noguchi, Kazuhiro Ooi, Shuichi Kawashiri

**Affiliations:** Department of Oral and Maxillofacial Surgery, Kanazawa University Graduate School of Medical Science, 13-1 Takara-Machi, Kanazawa, 920-8641 Japan

**Keywords:** Tongue squamous cell carcinoma, Late cervical lymph node metastasis, Mode of invasion, Depth of invasion, Perineural invasion

## Abstract

To determine the predictive indexes of late cervical lymph node metastasis in early tongue squamous cell carcinoma (TSCC). We retrospectively analyzed the cases of 25 patients with stage I/II TSCC who had undergone surgical treatment without elective neck dissection. We evaluated the relationships between clinicopathologic factors and the occurrence of late cervical lymph node metastasis. Of the 25 cases, metastasis to the cervical lymph nodes was observed in nine cases (36.0%). The clinicopathological factors associated with late cervical lymph node metastasis were the mode of invasion (MOI, *p* = 0.032), depth of invasion (DOI, *p* = 0.004), and perineural invasion (PNI, *p* = 0.040). A multivariate analysis revealed that only the DOI was an independent predictor of late cervical lymph node metastasis. The combination of the DOI and MOI or the PNI and MOI was significantly correlated with late cervical lymph node metastasis (*p* = 0.004 and *p* = 0.012, respectively). Our findings suggest that combinations of the MOI, DOI, and PNI could be used as an index for predicting late cervical lymph node metastasis in early TSCC.

## Introduction

Lymph node metastasis is one of the factors in cancer patients that is predictive of prognosis. Tongue squamous cell carcinoma (TSCC) is the most common oral cancer and is characterized by an extensive and well‐developed vascular and lymphatic system and a high rate of metastasis to cervical lymph nodes [1]. Patients with early TSCC generally achieve good outcomes by undergoing the surgical resection of primary sites, [2] but there some cases of stage I/II TSCC metastasize to one or more cervical lymph nodes after the surgical resection of the primary site and become difficult to control. It was reported that 27%-40% of cervical lymph node metastases in TSCC cases are found at an early stage [3] and that the probability of late cervical lymph node metastases in early TSCC ranges from 14 to 29% [4, 5].

As a method for detecting and evaluating cervical lymph node metastasis, both palpation and imaging examinations are widely used. A clinical examination along with imaging modalities such as magnetic resonance imaging (MRI), computed tomography (CT), ultrasound (US), and positron emission tomography (PET) have been used to detect nodal metastasis. Although the detection sensitivity of lymph node metastasis has been improved by the progress in imaging diagnoses, the detection rate of lymph node metastasis provided by the current imaging test is only ~ 70%, and it not yet possible to detect micrometastases [6, 7]. In order to improve the survival rate of TSCC, it is extremely important to predict and detect the micromeastases to cervical lymph nodes and to deal with them at an early stage.

Many pathological predictors, including immunohistochemical staining, have been studied for cervical lymph node metastasis of TSCC [8, 9], but sensitive and reliable predictors of cervical lymph node metastasis have not yet been identified. We conducted the present study to determine the relationships between the occurrence of late cervical lymph node metastasis and clinicopathological factors in patients with early TSCC, and we examined the effective factors that could be useful to predict late cervical lymph node metastasis in patients with early TSCC.

## Materials and methods

### Patients and tumor samples

Of the 29 cases of TSCC at stage I/II that were treated between 2008 and 2017 at Kanazawa University Hospital’s Department of Oral and Maxillofacial Surgery, we retrospectively analyzed the cases of the 25 patients who underwent surgery at the primary site as their initial treatment. Prior to the initial surgery, all of the patients were confirmed by contrast-enhanced CT, US, contrast-enhanced MRI and/or FDG-PET to be without cervical lymph node metastasis, distant metastasis, or multiple cancers of other organs. The Union for International Cancer Control (UICC) system (7th and 8th editions) was used for the TNM classification [10, 11]. The nine males and 16 females ranged in age from 30 to 84 years (mean 60.2 yrs, median 65.0 yrs). All patients were treated with a partial glossectomy without elective neck dissection in the first surgery. The follow-up duration ranged from 20 to 118 months (mean of 57.6 months). Ethical approval for the present study was obtained from the Ethics Committee of the Kanazawa University Graduate School of Medical Science and all methods were performed in accordance with relevant guidelines and regulations. Written informed consent was obtained from each patient.

### Clinicopathological parameters

We examined the relationships between late cervical lymph node metastasis and the following clinicopathological parameters: age, gender, clinical stage (7^th^ and 8^th^ UICC editions), histological grade, mode of invasion (MOI) as the index of the pattern of tumor invasion, depth of invasion (DOI), lymphovascular invasion (LVI), and perineural invasion (PNI). The histological grades were classified based on the World Health Organization (WHO) criteria. The MOI values were classified based on the YK classification reported by Yamamoto et al. [12] The DOI was measured as the vertical distance from the mucosal surface of the tumor to the deepest point of tissue invasion in millimeters, measured by experienced pathologists.

### Statistical analyses

JMP 13 software program (SAS, Cary, NC, USA) was used to analyze all data. Kaplan–Meier method was used to calculate the 5-year cumulative survival rates and differences were tested by the log-rank test. We used the Fisher’s exact test to examine the relationship between late cervical lymph node metastasis and each clinicopathologic parameter. For parameters with significant differences, multivariate analysis was performed using Cox’s multivariate proportional hazard model to investigate the parameter’s predictive values. The OR value was used to reflect the risk of late cervical lymph node metastasis. A probability value less than 0.05 was judged to be statistically significant.

## Results

### Patient characteristics

**Fig. 1:**
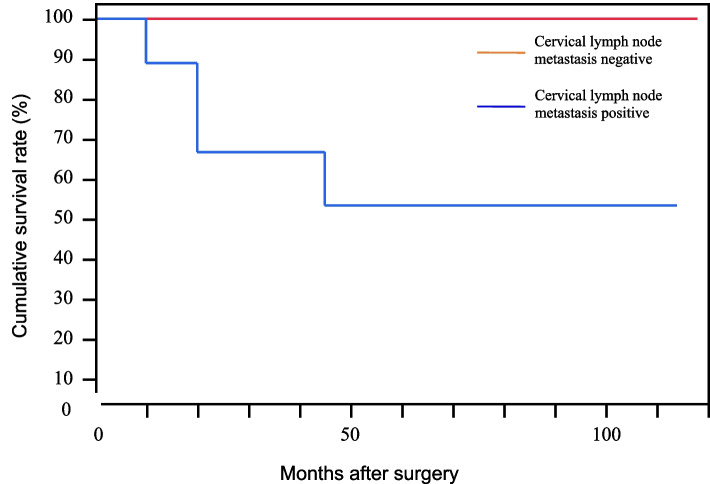
Kaplan–Meier estimates for 5-year cumulative survival based on late cervical lymph node metastasis. The 5-year cumulative survival rate of patients with late cervical lymph node metastasis (53.3%) was significantly lower than that of patients without it (100%) (*p* = 0.0043)

In all 25 patients, the excisional margin of the primary site was negative and local recurrence was not observed during the follow-up. Nine of the patients (36%) had late cervical lymph node metastases, and the average duration from local surgery to the discovery of cervical lymph node metastasis was 5.8 months. A receiver operating characteristics (ROC) curve indicated that the most accurate prediction of late cervical lymph node metastasis was made when the DOI cutoff value was 4 mm. The 5-year cumulative survival rate for the patients without late cervical lymph node metastasis was understandably 100%, whereas the 5-year cumulative survival rate for those with late cervical lymph node metastasis was 53.3% (Fig. 1), confirming that late cervical lymph node metastasis affects the 5-year cumulative survival rate (*p* = 0.043).

### The relationship between clinicopathological factors and late cervical lymph node metastasis

Our analyses of the relationships between clinicopathological factors and late cervical lymph node metastasis revealed, no significant differences between the groups with and without lymph node metastases in age, sex, clinical stage (7th ed.), clinical stage (8th ed.) or LVI, but they did reveal significant between-group differences in the MOI, DOI and PNI and late cervical lymph node metastasis (*p* < 0.05) (Table 1). The results of the multivariate analysis of the three factors with significant differences demonstrated that only the DOI was an independent predictor of late cervical lymph node metastasis (Table 2).

**Table 1:** Clinicopathological parameters in relation to late cervical lymph node metastasis in patients with TSCC

		Late cervical lymph node metastasis		
Parameter	n	+	-	*p* value
Age,yrs:				1.00
< 65	12	4	8	
≥ 65	13	5	8	
Sex				
Male	9	2	7	0.401
Female	16	7	9	
Clinical stage (UICC 7th Ed.):				0.411
T1N0	12	3	9	
T2N0	13	6	7	
Clinical stage (UICC 8th Ed.):				0.182
T1N0	8	1	7	
T2N0	17	8	9	
Imaging modalities (Total number):				0.519
CT	25	9	16	
US	4	2	2	
MRI	4	3	1	
FDG-PET	18	8	10	
Histological grade:				0.216
Grade 1	8	1	7	
Grade 2	10	4	6	
Grade 3	7	4	3	
MOI:				0.032
Type 1	2	0	2	
Type 2	5	1	4	
Type 3	11	4	7	
Type 4C	3	0	3	
Type 4D	4	4	0	
DOI:				0.004
< 4 mm	14	1	12	
≥ 4 mm	11	8	4	
LVI:				0.097
-	12	2	10	
+	13	7	6	
PNI:				0.040
-	20	5	15	
+	5	4	1

**Table 2:** Multivariate analyses of clinicopathological parameters in relation to late cervical lymph node metastasis in patients with TSCC

		***P*-value**		
Factors	**Group**	**Univariate**	**Multivariate**	**OR (95%CI)**
MOI	Other than type 4D/type 4D	0.032	0.1368	
DOI	< 4 mm/ > 4 mm	0.004	0.0286	12.299 (0.977–154.657)
PNI	-/ +	0.040	0.4378	

### The relationship between combinations of the MOI and DOI and late cervical lymph node metastasis

We examined whether a combination of MOI, DOI or PNI could predict late lymph node metastasis. We first investigated the relationship between the combination of MOI and DOI and late cervical lymph node metastasis (Table 3) and observed 55.6% sensitivity, 93.8% specificity, the positive predictive value 83.3% and the negative predictive value 79.0%. In addition, there were significantly fewer cases of late cervical lymph node metastasis among the cases with other than MOI type 4D or a DOI < 4 mm (*p* = 0.004).

**Table 3:** Relationship between the combination of MOI and DOI and late cervical lymph node metastasis

	**Late cervical lymph node metastasis**		
	+	-	**n**
**MOI type 4D or DOI ≥ mm**	8	4	12
**MOI other than type 4D or DOI < 4 mm**	1	12	13
**n**	9	16	25

### The relationship between combinations of the MOI and PNI and late cervical lymph node metastasis

We also evaluated the relationship between the combination of the MOI and PNI and late cervical lymph node metastasis (Table 4), and we observed 55.6% sensitivity, 93.8% specificity, the positive predictive value 83.3%, and the negative predictive value 79.0%. There were significantly more cases of late cervical lymph node metastasis among the cases with the combination of MOI type 4D and PNI-positive status (*p* = 0.012).

**Table 4:** Relationship between the combination of MOI and PNI and late cervical lymph node metastasis

	Late cervical lymph node metastasis		
	+	-	**n**
**MOI type 4D or PNI positive**	5	1	6
**MOI other than type 4D or PNI negative**	4	15	19
**n**	9	16	25

## Discussion

The question of whether elective neck dissection should be performed or a conservative observation approach should be taken for cervical lymph nodes in early-stage oral squamous cell carcinoma has been a matter of debate [13, 14]. It was reported that approx. 25% of patients with TSCC present with occult metastasis at their first medical examination [15, 16] and that not only local recurrence, but also cervical lymph node metastases affect overall survival [17–20]. Our present findings also suggest that late lymph node metastases affect overall survival. There are many reports that elective neck dissection is recommended if the risk of nodal metastasis is > 15%, [21–24] but there is another opinion proposing a conservative observation approach in which strict follow-up is performed in order to avoid unnecessary neck dissection. In any case, we believe that the predictive factors for the late cervical lymph nodes metastasis in early TSCC are important and necessary when considering elective neck dissection or as the conditions for strict follow-up by the conservative observation approach.

Many studies have searched for predictors of late cervical lymph node metastasis by using immunohistochemical techniques for primary tumors. The immunohistochemical expression patterns of pan-cytokeratin and podoplanin were reported to be an effective predictor of late cervical lymph node metastasis of TSCC [25]. A 2004 study attempted to predict late cervical lymph node metastasis by detecting vessels [26]. Such research requires complicated methods (e.g., immunostaining or specific staining), whereas only the common tissue stain hematoxylin–eosin was the only method needed in the present study. We also used the MOI as an index; the MOI was first reported by Yamamoto et al. [12] and is classified into five types. Type 4D, which pathologically has no distinct borderline between cancer and stroma and cancer cells diffusely invade, has the highest invasive tendency and metastatic ability [27]. In our present analyses as well, there was a significant difference between the MOI and the occurrence of late cervical lymph node metastasis (*p* = 0.03).

Many data have been published concerning the relationship between the DOI and cervical nodal metastasis with many studies emphasizing its role as a valid predictor [21, 24, 28–30]. O-charoenrat et al. used 5 mm as a cut-off in early oral tongue cancer and demonstrated that the 5-year survival was 95% with the tumor thickness of 5 mm and 30% when the tumor thickness was > 5 mm [31]. In the current UICC TNM classification (8th ed.), the DOI is incorporated into the T staging, and the DOI has been shown to be an important factor in redefining the staging system, resulting in up-grading based on the DOI cut-off of 5 mm and 10 mm [11]. Our present findings demonstrate that compared to the UICC 7th edition, the clinical staging of the UICC 8th edition shows late cervical lymph nodes metastasis in most cases of stage II, but the difference was not significant.

When we conducted the present investigation with the DOI cut-off set at 4 mm, a significant number of cases of late cervical lymph node metastasis were found among the cases with a DOI ≥ 4 mm. Moreover, our multivariate analysis of the association of the DOI and late cervical lymph node metastasis revealed that the cases with a DOI ≥ 4 mm were at a fourfold greater risk of developing late cervical lymph node metastasis compared to cases with a DOI < 4 mm. Balasubramanian et al. reported that the cervical lymph node metastasis rate was 11.2% in patients with a DOI < 4 mm and 38.5% in those with a DOI ≥ 4 mm, [32] and our data support their findings. Moreover, a cut-off point of 4 mm has traditionally been used to guide the decision for elective neck dissection, based on a study by Kligerman et al. [17]. These results suggest that even a tumor that has a DOI shallower than the cut-off value of 5 mm in the UICC 8^th^ edition may cause late cervical lymph node metastasis.

Other DOIs have been reported to be predictive factors of late cervical lymph node metastasis [33], and further validation of the past and present data is required. Herein we observed that when we combined the DOI and the MOI, in cases with a tumor other than MOI type 4D or with a DOI < 4 mm, the occurrence of late cervical lymph node metastasis was very rare (*p* = 0.004). Moreover, this combination provided 88.9% sensitivity, 75.0% specificity, the positive predictive value 66.7% and the negative predictive value 92.3%. Evaluating the combination of DOI and MOI may therefore be useful as a factor for identifying patients who do not develop late lymph node metastasis.

Many of the present patients with PNI had late lymph node metastasis (*p* = 0.012). Lymph node metastasis was observed in another study including patients with PNI, and the patients’ prognoses were poor [34]; we obtained a similar result. We observed that by combining the present of PNI and the MOI, there were very few cases of late lymph node metastasis among the patients with MOI type 4D or PNI (*p* = 0.002), and the combination provided 55.6% sensitivity, 93.8% specificity, the positive predictive value 83.3%, and the negative predictive value 79.0%. Therefore, evaluating the combination of PNI and the MOI may be useful as a predictive factor of late cervical lymph node metastasis.

Our study had several limitations. First, this study was based on a small sample size because the incidence of oral cancer is 1.6% of all malignant neoplasms [35], which is not so common, and this study targeted early TSCC without cervical lymph node metastasis at the time of their initial treatment. Second, because our study was a retrospective cohort, there could be missing data. Eventually, despite these limitations, this study provides an interesting relationship between the pattern and spread of invasion in early TSCC and late cervical lymph node metastases.

## Conclusion

By examining the combination of MOI and DOI or PNI, we believe that it will be possible to more reliably predict the development of late cervical lymph node metastasis. The present patients who had MOI type 4D, a DOI ≥ 4 mm, or PNI are considered being at high risk of late cervical lymph node metastasis, and elective neck dissection should be considered. If a conservative observation approach is to be taken for such patients, strict follow-up including periodic image examinations is essential, and if cervical lymph node metastasis is suspected, an immediate response should be taken, and it is expected that the prognosis of TSCC can be improved.

## Data Availability

The raw data supporting the conclusions of this article will be made available by the authors.
